# A Deep Learning Approach to Predict Chronological Age

**DOI:** 10.3390/healthcare11030448

**Published:** 2023-02-03

**Authors:** Husam Lahza, Ahmed A. Alsheikhy, Yahia Said, Tawfeeq Shawly

**Affiliations:** 1 Department of Information Technology, Faculty of Computing and Information Technology, King Abdulaziz University, Jeddah 21589, Saudi Arabia; 2 Department of Electrical Engineering, College of Engineering, Northern Border University, Arar 91431, Saudi Arabia; 3 Department of Electrical Engineering, Faculty of Engineering at Rabigh, King Abdulaziz University, Jeddah 21589, Saudi Arabia

**Keywords:** age prediction, CNN, human eyes, face recognition, color intensity, vision 2030

## Abstract

Recently, researchers have turned their focus to predicting the age of people since numerous applications depend on facial recognition approaches. In the medical field, Alzheimer’s disease mainly depends on patients’ ages. Multiple methods have been implemented and developed to predict age. However, these approaches lack accuracy because every image has unique features, such as shape, pose, and scale. In Saudi Arabia, Vision 2030, concerning the quality of life, is one of the twelve initiatives that were launched recently. The health sector has gained increasing attention as the government has introduced age-based policies to improve the health of its elderly residents. These residents are urgently advised to vaccinate against COVID-19 based on their age. In this paper, proposing a practical, consistent, and trustworthy method to predict age is presented. This method uses the color intensity of eyes and a Convolutional Neural Network (CNN) to predict age in real time based on the ensemble of CNN. A segmentation algorithm is engaged since the approach takes its input from a video stream or an image. This algorithm extracts data from one of the essential parts of the face: the eyes. This part is also informative. Several experiments have been conducted on MATLAB to verify and validate results and relative errors. A Kaggle website dataset is utilized for ages 4 to 59. This dataset includes over 270,000 images, and its size is roughly 2 GB. Consequently, the proposed approach produces ±8.69 years of Mean Square Error (MSE) for the predicted ages. Lastly, a comparative evaluation of relevant studies and the presented algorithm in terms of accuracy, MSE, and Mean Absolute Error (MAE) is also provided. This evaluation shows that the approach developed in the current study outperforms all considered performance metrics since its accuracy is 97.29%. This study found that the color intensity of eyes is highly effective in predicting age, given the high accuracy and acceptable MSE and MAE results. This indicates that it is helpful to utilize this methodology in real-life applications.

## 1. Introduction

Changes in the brain volume and its functionalities are associated with aging. Some diseases, such as Alzheimer’s, are related to changes in brain volume and atrophy [1,2]. Thus, it is possible to estimate age based on brain volume measurements. Researchers identify two types of ages, biological and chronological [2]. Recently, studies have shown that a person older chronologically than biological ages more slowly than expected. In the Machine Learning (ML) field, age prediction refers to brain age using a regression model [1,2]. In the industrial area, facial recognition systems and applications based on deep learning approaches are widely used and adopted because of their high accuracy [3]. The government of Saudi Arabia is expected to spend over 25 billion US dollars on artificial intelligence systems and applications; among those systems is facial recognition, which will be used to facilitate Saudi government regulations and rules. Nevertheless, those systems have shown poor accuracy in numerous cases [3,4,5]. IBM has shut down its facial recognition-based applications due to their inadequate accuracy [3]. Several large enterprises and technology leaders, such as Microsoft and Amazon, have restricted their systems for use only in law enforcement organizations [3]. Biological brain age has been utilized as a quantitative index for analyzing brain health for the entire lifespan [6]. Several studies predicted brain age using extensive neuroimaging methods from healthy participants [6]. In addition, these studies depicted potential relations between brain age and physical activity, mortality risk, and grip strength [6].

Several deep-learning analytical frameworks have been proposed as end-to-end solutions [6,7]. Compared with older approaches, these solutions have provided a higher estimation accuracy [6]. In addition, various image preprocessing procedures and feature extraction were omitted by those models [6]. However, these preprocessing and feature extraction procedures depend on the utilized quality of the studied images and the selected software [6]. Researchers have recently focused on finding a relationship between modalities of brain MRIs and brain tissue properties to predict age. An ensemble learning algorithm is an effective Machine Learning (ML) approach to reaching higher performance [6,7,8]. This model has reduced overfitting while improving performance [6,8,9]. In past years, numerous methods to estimate age were implemented. Hand-crafted feature extraction from facial images was the first proposed work to predict age [9,10]. Neural Network (NN) models have recently been utilized in most developed algorithms due to their higher performance [9]. A CNN model is employed and used in numerous solutions to estimate age due to its positive reputation for feature extraction [10,11,12].

**Figure 1:**
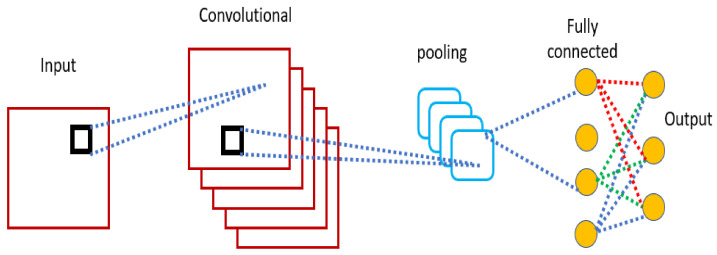
A typical CNN structure.

A Convolutional Neural Network (CNN) is a deep learning approach that takes an input or a sequence of inputs through different levels to produce outputs. Figure 1 illustrates a typical CNN architecture.

A CNN typically contains five convolutional layers, as depicted in Figure 1. Generally, the typical CNN can be seen as four blocks: input, convolutional, fully connected, and output, as illustrated in Figure 1.

Estimating or predicting age is challenging since the number of predictable age groups is higher than expected [3,4,5,6]. Numerous law enforcement, marketing, and security applications use age prediction in their functions [3]. Age prediction is one of the biological profiles utilized in forensic investigation and analysis based on shoeprint methods [4]. Recent studies by the United Nations Department of Economic and Social Affairs predicted that by 2050, one out of six people is estimated to be over 65 [5]. In addition, a progressive decline in physical abilities relates to aging [5]. Thus, it is critical to estimate age using a reliable approach. Numerous machine learning and artificial intelligence approaches utilize images, videos, and MRIs to predict biological ages [5,6,7]. Multiple applications in different fields, such as marketing and biometrics, rely on age information to gain customers and increase profits.

**Figure 2:**
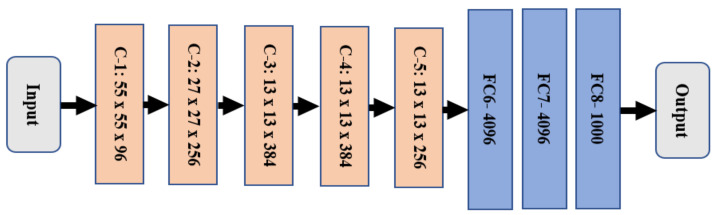
AlexNet architecture.

An AlexNet, a type of CNN tool, is utilized for this profound learning purpose. This model typically consists of eight layers, as illustrated in Figure 2. These eight layers are generally categorized into two layers which include five convolutional layers, denoted in light orange, and three fully connected layers, marked in blue.

AlexNet utilizes Rectified Linear Units (ReLU) to speed up execution [13,14]. It consists of filter sizes: 11 × 11, 3 × 3, and 5 × 5. AlexNet takes an input of size 227 × 227 × 3 to process it. The size is then shrunk into smaller sizes, such as 55 × 55 × 96, 27 × 27 × 96, and 13 × 13 × 256. In addition, two dropout layers are utilized [15,16,17,18,19].

### 1.1. Problem Research

This current study presents a method to predict chronological age using the color intensity of human eyes and a deep learning approach. To the best of our knowledge, this is the first study to indicate the chronological age using eyes. The current existing methodologies use different features and instances to evaluate the age, and these methods lack accuracy, as the maximum achieved accuracy was nearly 90%. Therefore, the presented algorithm estimates age precisely based on the color intensity of the human eye, and its accuracy is over 96% according to the simulation results.

### 1.2. Research Objective

This research aims to present, develop, and implement a novel algorithm to predict age precisely and appropriately. The actual age labels feed this algorithm to improve its accuracy. In addition, this model computes several performance metrics to compare the proposed method with other state-of-the-art models.

### 1.3. Research Motivations and Contributions

The motivations for this research stem from a need to have a feasible method to predict age quickly and accurately. In the Saudi 2030 vision, the government of Saudi Arabia launched a promising vision in several areas, such as quality of life, the economy, and security. Developing a dependable and viable approach to predict age precisely and accurately is the motivation for this research due to the rapidly increasing need in different areas and applications. Furthermore, many researchers in other fields, such as public health and sociology, have used public data from social media to understand users’ behavior and assess their medical and health conditions; however, their effort is limited by the lack of critical demographic indicators as age [20]. Therefore, it is crucial to have a flexible method for predicting an individual’s age from publicly available data. Having precise results of age prediction is required for applications where high accuracy is necessary.

In this paper, the contribution is achieved by developing and proposing an algorithm to estimate chronological age based on the color intensity of human eyes. This approach is implemented using MATLAB as a programming platform and simulation tool. The proposed method uses the human eye-based system to extract color intensity from images or videos to predict chronological ages in real time. This algorithm is trained in a multitask learning classifier. The extracted features, such as peaks and bottom values of the color intensity, are embedded into the classifier to increase and improve the obtained accuracy. This model uses images from the Kaggle dataset for training and validating its outputs. The rest of the paper is organized as follows: a literature review of related research is presented in Section 1.4. Section 2 provides details on the materials and the approach to developing this method. Results are provided in Section 3, followed by a discussion in Section 4, and the conclusion is given in Section 5.

### 1.4. Related Work

In healthcare, medicine, and medical sociology, chronological age prediction has been widely used in research and practice to categorize and group individuals. This section will contextualize the proposed work concerning the difficulties in predicting a person’s age for medical and behavioral diagnosis and treatment and present a deep learning approach as a state-of-the-art solution concerning the different methods literature for age prediction.

#### 1.4.1. Healthcare Perspective

Patients’ ages are one of the key demographic indicators that are widely used in practice and research. For example, during the COVID-19 pandemic, patients were prioritized and categorized based on their age for treatments and diagnostics. Age is frequently used as a primary criterion for allocating scarce medical resources during pandemics [21]. Chronological age is defined as a person’s official age from the day of birth to the present. It is used to predict the age of individuals or tissues and can be effectively used to predict, assess, and evaluate various types of diseases [22]. Biological age is also referred to as the age that can indicate the decline in health and physical function throughout a person's lifespan [23]. Both concepts, chronological and biological age, have been used in numerous studies in the medical field, in which participants give the former, and the latter is predicted using different biological factors, such as epigenetic clocks [23,24,25,26,27]. Recently, many research studies in the medical field have used publicly available data to monitor the spread of diseases, evaluate behavioral changes in certain populations, and assess their attitude toward health issues and trends. Nevertheless, these efforts were limited by the lack of key demographic indicators, such as chronological age [20]. This research study provides a state-of-the-art deep learning approach to predicting the chronological age of individuals from photos and videos using the color intensity of the eye.

A. Zaguia et al. in Ref. [1] developed a model to estimate age based on DNA methylation biomarkers using healthy and diseased samples. A public dataset with 854 images was utilized, and it was publicly available. This dataset contained ages from 1 to 89 years old and split into 80:20 ratios for training and testing purposes. The authors used four machine-learning techniques. The authors aimed to infer the diseases that affect age adversely. Three performance metrics were evaluated: the coefficient of determination (R2), RMSE, and MAE. The developed algorithm achieved 4.85 and 9.53 of MAE for healthy and diseased samples, respectively. In contrast, the proposed model trained over 100,000 images, and it achieved 2.43 years old of MAE, which is better than what has been reached in Ref. [1]. In addition, the presented method used one deep learning technique instead of four, as in Ref. [1]. The proposed algorithm can be utilized to predict diseases that are related to age, such as Alzheimer’s disease, Parkinson’s disease, and stroke, after some adjustments.

In Ref. [2], the authors evaluated three datasets using 27 machine-learning techniques to predict brain age. These datasets contained 2281 MRI images. The implemented model achieved 2.75–3.12, 7.08–10.50, and 8.04–9.86 years on all datasets, respectively. The authors were targeting the relationship between age and disorders of diseases that affect humans, such as mental health. There was no novelty in Ref. [2], as the authors only evaluated 27 models, while the presented model uses the color intensity of eyes to estimate age. This estimation can help predict various diseases that are related to age. In addition, the utilized dataset contains more than 100,000 images, while the authors in Ref. [2] used only 2281 images. Furthermore, the proposed method achieved 2.43 years of age for MAE, which is lower than what the authors in Ref. [2] reached.

#### 1.4.2. Technical Perspective

H. Hobday et al. in Ref. [3] developed and implemented an approach to predict age based on rapid structural brain scans. The authors compared conventional EPImix-derived T1-weighted scans from more than 50 healthy participants to estimate the tissue volume and age of the brain. All operations were carried out based on an SPM DARTEL pipeline method. The authors claim that their approach reduced scanning time on the brain and cost while maintaining participants’ comfort. Readers can refer to Ref. [3] for more information. The proposed algorithm in this article uses human eyes through deep learning to estimate age based on the color intensity of the detected eyes. It is cost-effective since there is no need to scan the brain, as a real-time image or a video recording can adequately predict age.

J. G. Ramirez et al. in Ref. [4] used approximately 3500 MRIs to analyze changes in the brain to predict chronological age only. Automated brain segmentation and parcellation methods were utilized on healthy individual elders between the ages of 69 and 88. The authors obtained a Mean Absolute Error (MAE) of 2 years in age estimation when applying this method to new participants. Interested readers can refer to Ref. [4] for more information. Herein, the proposed algorithm is utilized to estimate the chronological age. Its accuracy is nearly 90%, while the obtained MSE is less than nine years.

In Ref. [5], Y. Cao et al. implemented a method to estimate age using distribution-aware data curation and augmentation using a Deep Neural Network (DNN) tool on facial recognition systems. The developed method achieved 4.92 times fairness compared with other approaches. The IMDB-WIKI dataset was used to train and validate the model for four benchmarks. These benchmarks were APPA-REAL, MORPH-2, UTKFace, and Mega Asian. MAE was determined to be used as a performance metric. All tested benchmarks gave a different value for MAE, and the maximum obtained value was 7.27 years, while in this article, the presented algorithm reaches approximately ±8.7 years of MSE. Readers are referred to Ref. [5] for more information.

M. Hassan et al. in Ref. [8] developed a method to predict age from shoeprints using a deep analysis technique. Over 100,000 samples of shoeprints were collected from participants from 7 to 80 years old. The authors named their model ShoeNet to analyze factors and patterns related to age estimation. This model utilized CNNs based on a skip mechanism to extract features that were related to age. However, the obtained errors were nearly within five years. In addition, the maximum obtained MAE was 9.21 years of age. More information can be found in Ref. [8].

S. Xu et al. in Ref. [11] improved the accuracy of age estimation based on an AutoML model. The authors utilized AutoML from Google Cloud as a training tool. Approximately 4900 facial images were used. These images were of Asian people and were categorized into six classes based on the ages of the participants. This model achieved nearly 69.83% precision when changing the threshold from 0.5 to 0.71. It was good at differentiating ages from 10 to 40, but it was poor with those outside this range. Hence, this model was suitable for young participants only. In contrast, the developed algorithm applies to children, youth, and elderly participants. Interested readers are referred to Ref. [11] for more information.

In Ref. [13], K. Stankeviciute et al. presented a method to predict brain age using Population Graph GNNs. Two types of GNN architectures were trained to estimate age clinically. A United Kingdom Biobank (UKB) dataset was utilized and tested. It had MRIs from over 500,000 participants. The authors used MSE to validate their model, and each type achieved different values. The maximum obtained MSEs were over 26, while our algorithm achieves nearly 8.7 of MSE, which outperforms the presented model in Ref. [13]. Readers can refer to Ref. [13] for more information.

A. Abdolrashidi et al. in Ref. [14] implemented a method to estimate gender and age using an Attentional Convolutional Network (ACN) based on facial images. The authors claimed that their model achieved promising results. A dataset of UTK-Face was utilized after modification. It had approximately 10,000 facial images for both genders and reached nearly 96% accuracy. However, it gave results in ranges rather than in specific numbers, while the algorithm proposed in this paper provides detailed results for the estimated ages.

In Ref. [17], A. Abu Nada et al. implemented a method to estimate age and gender through single-user images based on a CNN tool. The validation of a user’s age and gender was performed using the CNN method. In addition, a web service for validation was also implemented at the University of Palestine in Palestine. This method achieved nearly 82.24% accuracy, while the presented approach reached 97.29%. The proposed model produces promising results which are better than what was developed in Ref. [17]. Readers are referred to Ref. [17] for additional information and details.

## 2. Materials and Methods

### 2.1. Problem Statement

Some studies, such as those in Refs. [3,4,5] focused on brain scans to predict chronological ages, while [6] estimated ages based on the shoeprint. The studies [3,4,5] lack accuracy since it was less than 92%, while the research in Ref. [6] achieved less than 96% accuracy. We searched for studies that estimated ages based on the human eye but have not yielded any results. However, one study utilized facial images to predict ages with an accuracy of nearly 91%, and this study can be found in Ref. [11]. Hence, the intention is to have a reliable algorithm to predict the chronological ages accurately over what has been developed already. In addition, this method focuses on the human eyes to analyze their color intensity and estimate the ages. This algorithm should close the current gap accuracy. Furthermore, this research aims to have an acceptable range of execution time for age prediction in a quick manner.

The main goal of the proposed algorithm is to predict chronological age from the human eyes based on color intensity. The prediction is performed and calculated according to the obtained values of color intensity and the extracted required features. Age prediction is considered a challenging task since numerous factors play a significant role. These factors include the color of the eyes, genetics, and hormones.

### 2.2. Dataset

The utilized dataset was downloaded from the Kaggle website. It was separated into two groups: one group for training purposes representing 70% of the dataset, while the other was used for validation and testing purposes, as 15% has been allocated equally to each.

### 2.3. The Proposed Model

The presented algorithm predicts age based on human eye colors using CNN. The AlexNet model, a type of CNN, is utilized for training the approach deeply based on extracted features that are believed to be related to age, such as color and tissue inside the eyes. In this research, the colors of the eyes are considered. In the presented model, ten features are extracted and utilized. In total, 2,700,000 characteristics from the dataset are used. As stated earlier, the dataset used herein was downloaded from the Kaggle website and includes over 270,000 images. These images represent participants, from young to older people, from ages 4 into the late 50s. The training dataset includes approximately 189,000 photos and is categorized into four groups. Every group has its considered ages, several relevant samples, and the dimensions of the images, as listed in Table 1.

It was crucial to feed the approach with valuable features to train the proposed algorithm in a reliable mode. This was achieved by enhancing the quality of the samples through the built-in functions in MATLAB. This stage is performed using image processing techniques. All input samples are processed and grouped according to their pressure distributions. This is achieved by using a superimposed method in MATLAB. The superimposed approach refers to placing an image on top of others to reflect the obtained effect [4]. Every input is processed to find the contour and determine the corresponding bounding box coordinates. The inputs are then cropped according to the corresponding bounding box coordinates to begin analyzing the colors of the eyes. The mean age of the training dataset was 37.3 years for men and women, while the standard deviation was 14.8 years. The training dataset ran on a machine with 4 CPUs, 2.4 GHz, and 16 GB RAM. Total execution time and processing time in this research were recorded and stored for each distinct run. In total, 13 distinct runs were utilized to achieve higher accuracy. All 13 distinct runs lasted for nearly 9 hrs for training only.

Quantitative measurements are derived from accuracy, MSE, and MAE. In addition, we investigated the advantage of using a different regression stage to optimize the age prediction and estimation from the training dataset. We then performed the K-Fold Cross-Validation technique to verify the proposed algorithm. K was set to 5, as depicted in Table 2 since the minimum of K in MATLAB is 5. Every testing fold is distinguished in light blue, while the training fold is illustrated in light green.

The Viola-Jones algorithm is used and employed to detect faces and then the eyes of the input images. This approach was implemented in 2001 [28,29,30,31,32]. It is an object detection approach, and its primary function is to detect faces. In addition, it can detect different objects if it is appropriately trained. This Viola-Jones algorithm consists of two characteristics that make it a reliable approach to detection. These characteristics are as follows [30,31,32]:

1. A Real-Time method, since it processes two frames per second.
2. It is robust since its actual positive rate is higher than the false-positive rate.

In addition, four stages are involved: Haar Feature Selection, Creating an Integral Image, Adaboost Training, and Cascading Classifiers [30]. Readers can refer to [30] for additional details.

Object detection is one of the computer technologies employed in computer vision applications [32]. It is utilized to detect numerous objects, such as the whole face or parts of it, in addition to trees, cars, and buildings [32]. The Viola-Jones algorithm has proven to be a powerful tool for detecting faces in real time. However, its performance in the training stage is prolonged. In addition, it works on the grayscale level of inputs. Furthermore, it detects several faces inside any input by dividing it into numerous subregions and checking through them [31,33].

In image processing techniques, the grayscale level is used to measure the intensity, which is defined by the energy of light colors [33,34,35,36,37]. The brightness is considered a measure of intensity values generated from all pixels in any image [34]. The intensity in this research represents the light energy reflected by objects or transmitted through these objects.

**Figure 3:**
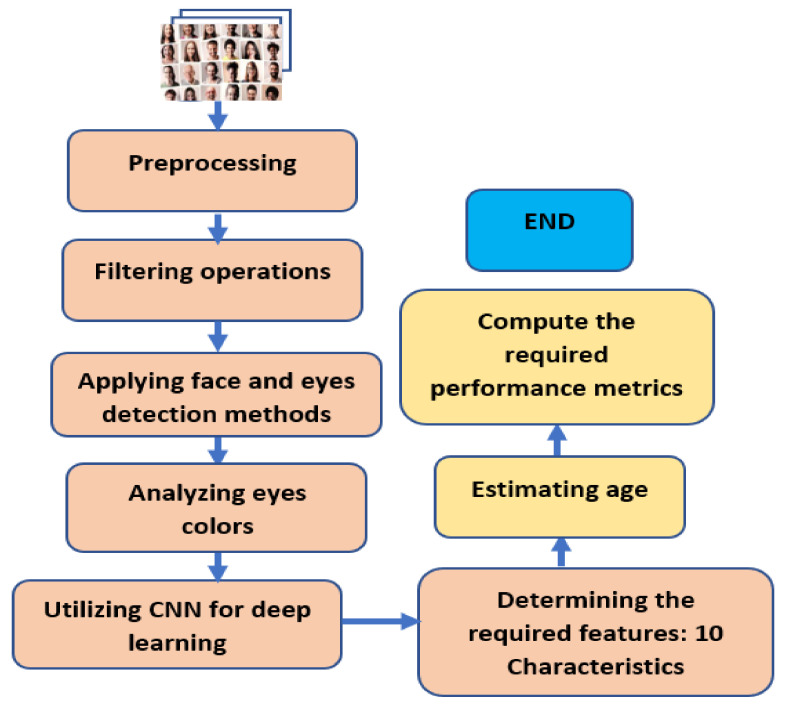
Flowchart of the presented algorithm.

The implemented model starts by reading an image or numerous images from a file or from a video CAM to provide real-time data. Preprocessing procedures are then performed to remove noise, resize the inputs and convert these inputs into equivalent gray images. Discrete Wavelet Transform (DWT), Principal Component Analysis (PCA), and morphological tools are utilized in this stage. After that, numerous built-in filters inside MATLAB are used to enhance the pixels. Black pixels are removed from every image after validation with a dynamic threshold generated in the image preprocessing stage. The Viola-Jones Algorithm is used to detect a face for every input. Later, eyes are detected and surrounded by a blue rectangle for each input, as illustrated in Cases 1 to 6. Once the eyes are detected, the images are cropped to remove unneeded areas using a built-in function inside MATLAB. Additional information about the Viola-Jones algorithm can be found in Refs. [28,29,30]. The detected eyes are then analyzed to determine colors to perform the deep learning technique using CNN to extract the required features. These colors are analyzed using a histogram tool, colormap tool, and indexed images to scale their true colors and map them to look up the actual colors. The standard deviation feature is extracted and considered along with other components in this article. Lastly, age estimation is performed utilizing the extracted feature. Figure 3 depicts a flowchart of the presented model, and the pseudo-code of the proposed algorithm, Algorithm 1, is demonstrated as follows:
**Algorithm 1: Determine human eye color intensity and age estimation****Input: a sequence of images or an image.** **Output: the predicted age or ages.** 1.**Read a sequence of images or an image.**2.**In the image preprocessing stage, Perform:**3.  Apply various operations, such as filtrations, DWT, and PCA, to remove noise, resize and convert inputs to gray ones.4.  Perform several filtration processes on inputs to enhance pixels.5.**End of the image preprocessing stage.**6.Perform the Viola-Jones method to detect a face, then eyes.7.**In the deep learning stage, Do:**8.  Analyze detected colors of eyes and perform deep learning using CNN.9.**  For *i* = 1: the size of the processing images**10.**    For *j* = 1: the size of the processing images**11.      Remove unwanted pixels outside the borders of the detected eyes.12.      Compute pixels sum to the standard deviation.13.      Determine a mean of standard deviation for color values.14.**    End**15.**  End**16.**End of the deep learning stage.**17.Predict ages and display computed outputs.18.Calculate the required performance metrics: accuracy, MSE, MAE, precision, recall, and F-score.19.**End of algorithm**

Several performance metrics are calculated in the developed algorithm, including TP, FN, FP, accuracy, MSE, MAE, precision, recall, and F-score.

Precision (Pr) is calculated as in Equation (1):

Pr = TP/(TP + FP)(1)

The quantity of recall (Rc) is computed as shown in Equation (2):

Rc = TP/(TP + FN)(2)

F-score is calculated as illustrated in Equation (3):

F-score = 2 × [(Pr × Rc)/(Pr + Rc)](3)

The presented algorithm in this research has several advantages, which are summarized as follows:

A. It is easy to use and maintain.
B. It is a trustworthy and consistent approach.
C. It is intelligent, as the algorithm learns by itself.
D. It produces and achieves promising results since its accuracy is more than 97% and its MSE is approximately ±8.7 years for 12,150 images.

## 3. Results

**Figure 4:**
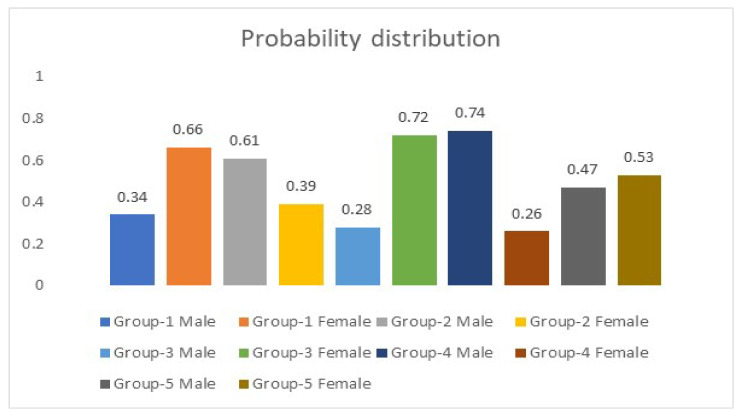
The computed probability distribution.

Five simulation experiments were conducted on numerous images to investigate the relationship between the extracted features from the eyes’ color intensity to predict ages to verify the model and evaluate its correctness. These experiments were carried out on a machine running on a Windows platform. Its specifications are an I7 Intel chip of the 8th generation. It is a 64-bit-based processor. The presented algorithm has been trained for over 10,000 iterations, the total number of epochs was 25, and it took nearly 9 h to complete. The learning rate is set to 0.0001 to optimize the loss function inside MATLAB. In addition, the presented algorithm computes the probability of the predicted ages for every group that is listed in Table 1. These probabilities refer to the gender of participants in every categorized group. Figure 4 represents a sample of the computed probability scores by the algorithm for every group in Table 1.

Table 3 shows the actual ages and genders of the sample inputs to the algorithm. In addition, each input is referred to as a case followed by its corresponding number. In this article, six cases are illustrated.

**Figure 5:**
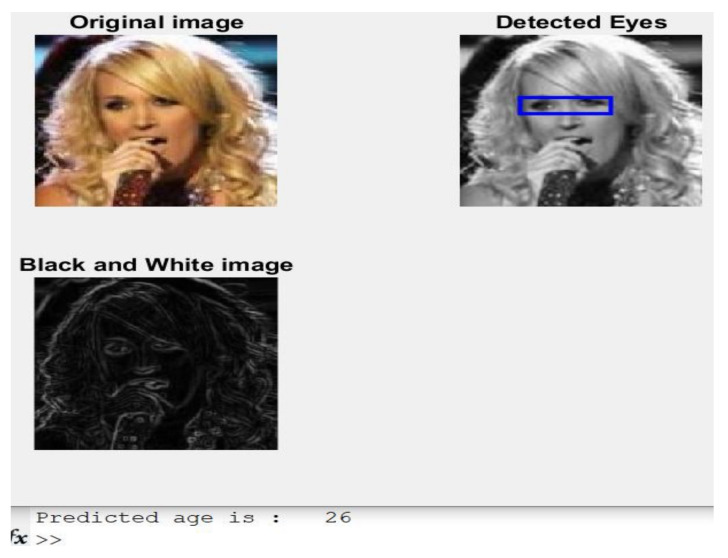
Results of Case 1.

**Case 1:**Figure 5 shows an image of a 26-year-old girl and her predicted age based on the presented model.

The presented approach predicted the exact age, which is 26. The implemented algorithm detects features from eyes such as color intensity, peaks, and bottoms corresponding to values, mean, standard deviation, and maximum and minimum values of color intensity. At the same time, the detected eyes are surrounded by the blue rectangle, as illustrated in Figure 5. The same concept is applied to all other cases to predict age.

**Figure 6:**
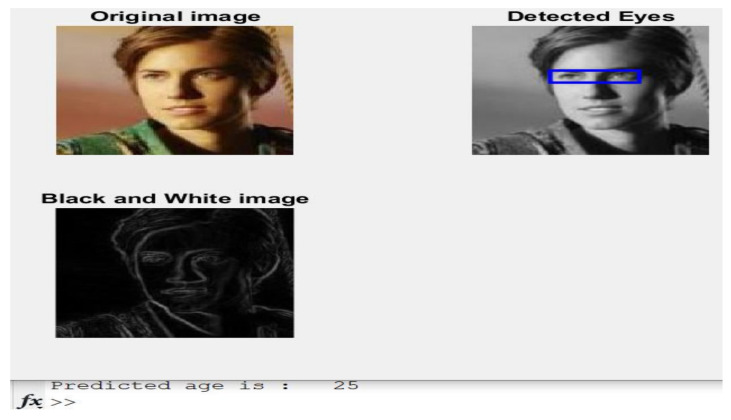
Obtained results of Case 2.

**Case 2:**Figure 6 displays another image of a 26-year-old girl and her estimated age.

The estimated age is 25, while the actual age is 26, as listed in Table 1. Furthermore, the presented algorithm is close enough to reach the exact age mentioned in Table 1.

**Figure 7:**
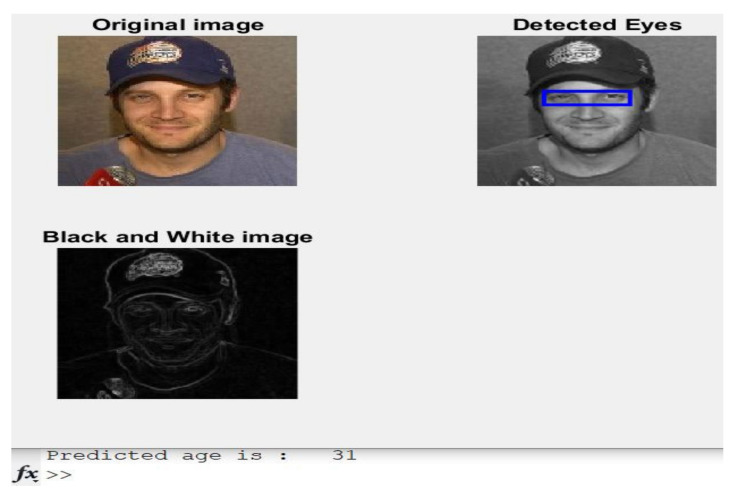
Obtained results of Case 3.

**Case 3:** A 32-year-old male is shown in Figure 7 with his predicted age.

The actual age was 32 years old, and the estimated age by the developed approach was 31, as shown in Figure 7.

**Figure 8:**
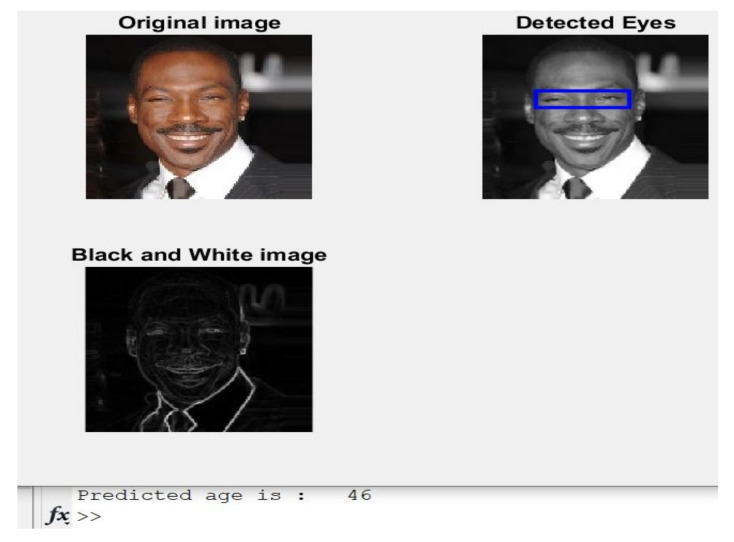
Obtained results of Case 4.

**Case 4:**Figure 8 depicts a picture of a 46-year-old male with his estimated age.

The implemented algorithm in this article predicted age correctly and accurately, as the expected age is 46 years, which is the same as the actual age listed in Table 1.

**Figure 9:**
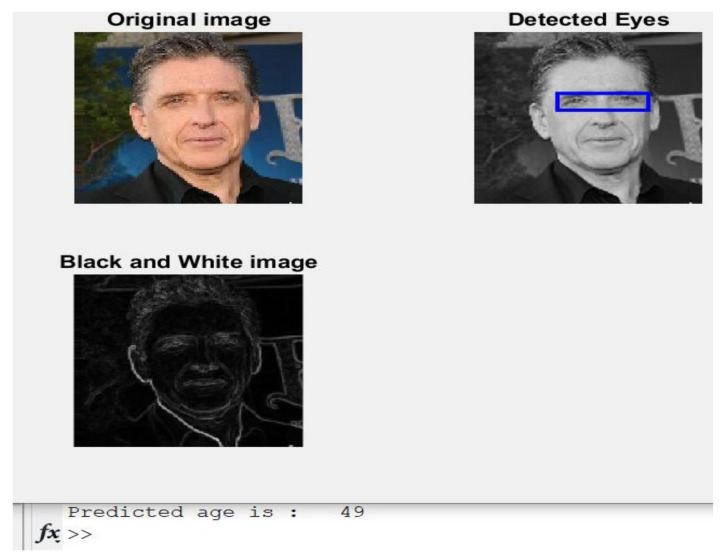
Obtained results of Case 5.

**Case 5:** An image of a 50-year-old male is shown in Figure 9 with the predicted age.

The presented approach was near the actual age since the estimation was 49 years old, and the exact age was 50.

**Figure 10:**
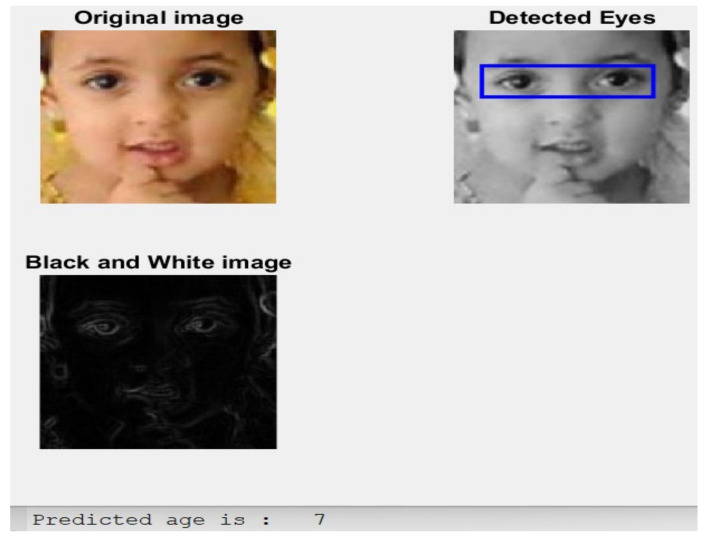
The obtained result of Case 6.

**Case 6:** an image of a five-year-old female child is shown in Figure 10 with the predicted age.

The implemented model was near the actual age since the estimated age is seven, while the exact age is 5. Thus, this approach produces promising results, as illustrated and shown in the previous six case studies.

Table 4 shows the obtained MSE and MAE for six cases. However, when applying the algorithm to a sequence of 12,150 images representing the testing dataset, the calculated MSE was nearly ±8.7 years, and the MAE was ±2.43 years.

**Figure 11:**
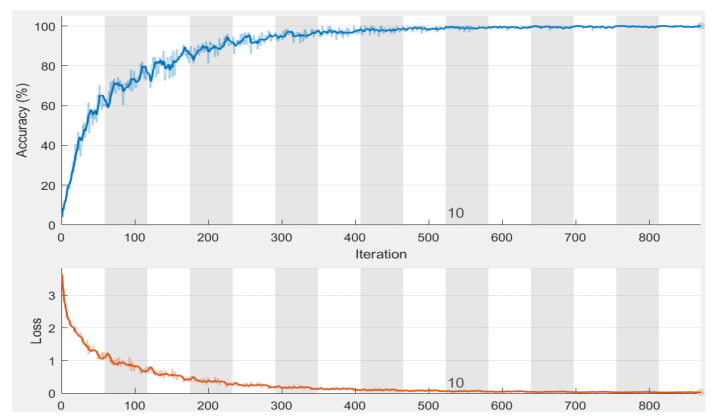
Sample of obtained accuracy.

**Figure 12:**
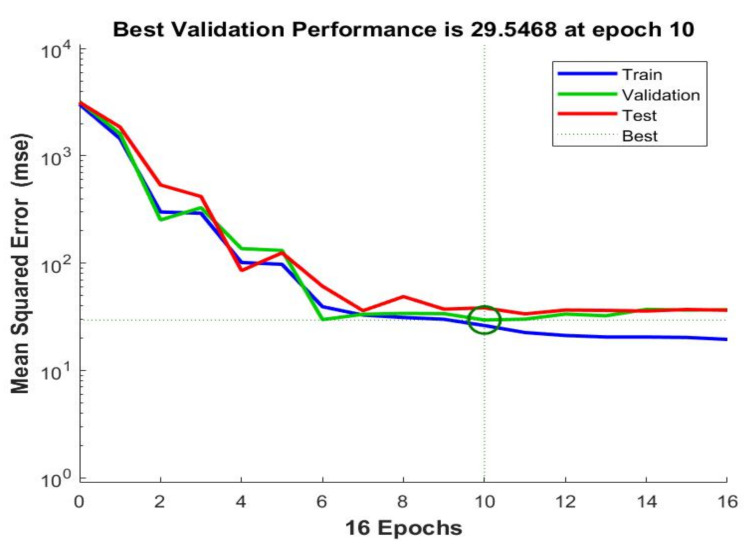
The obtained MSE chart of 16 epochs.

Figure 11 illustrates the convergence of accuracy of the training dataset for 15 epochs, along with the loss function. There were 870 iterations in total, and every epoch included 58 iterations. The obtained accuracy was 98.76%, and the loss converges to almost zero. Figure 12 shows the chart of MSE for 16 epochs when applied to the algorithm on 150 images. The best value occurred at 10 epochs with a value = 29.5468. This value decreases to less than 9 when using 85 epochs. The results were consistent with recent studies and research showing that no overall hazard could affect the associated features involved in estimating the chronological age.

**Figure 13:**
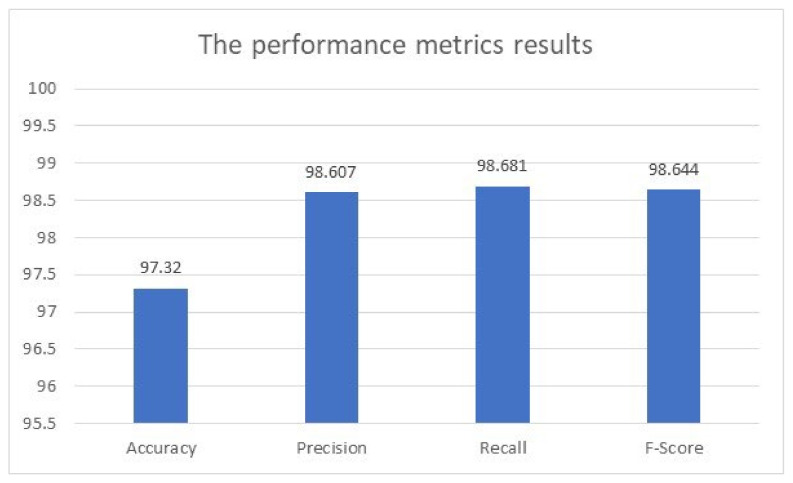
The achieved performance metrics results.

Table 5 shows the age estimation results of the considered performance metrics for 85 epochs. The performance was evaluated on a testing dataset of 12,150 images. Figure 13 illustrates the obtained performance metrics of accuracy, precision, recall, and F-score by the presented model when applied to the testing dataset.

Table 6 lists the comparison and evaluation results of the accuracy, MSE, and MAE between the developed algorithm herein and some studies referenced in Section 2. The proposed algorithm surpasses other state-of-the-art methods, as shown in Table 6.

The presented algorithm achieves a 5.45% improvement in MAE compared to [2]. This percentage represents a median value. The results in Table 6 indicate that the average value of the MAE is less than two and a half years, which is better than any conducted study in the literature mentioned in Section 2. The maximum results of MSE and MAE were 13.72 years and 4.09 years, respectively.

Table 7 lists the attained results from applying the implemented model to see which ages from the dataset have a higher or lower performance metric. The considered performance metrics are accuracy, precision, and F-score. The utilized dataset was divided into five categories. These groups are 4–19 years old, 20–29 years old, 30–39 years old, 40–49 years old, and 50–59 years old.

Table 8 shows a confusion matrix of the proposed model to predict ages on the testing dataset. This matrix shows the actual number of ages that have been estimated accurately and the total number of mispredictions.

## 4. Discussion

It is assumed that predicting chronological age from the eyes is possible by extracting the related features and estimating the ages of several test cases. The results of the conducted evaluation show a strong correlation between the extracted features from the eyes used to evaluate the chronological age. Previous state-of-the-art approaches were assessed, and it was observed that the obtained results needed to be more sufficient in terms of accuracy, MSE, and MAE. Figure 12 indicates that the values of MSE and MAE decrease as the number of iterations and epochs increases. This figure implies that the proposed model works perfectly and that its results are accurate and precise. Moreover, the characteristics of the training dataset can lead to different computations of MSE and MAE in age prediction, as some studies focus on a wide range of ages. In contrast, others have a limited range of ages.

The performed analysis on the obtained results of the implemented algorithm shows that it generates promising results, as its accuracy is 97.29%. In addition, this algorithm achieves the minimum MSE and MSE compared with other state-of-the-art methods, as shown in Table 6. It should be noted that the proposed model estimates chronological age. In contrast, biological age can be predicted using bio-features, which will be the next direction of this research. The proposed algorithm has been trained on more than 150,000 images to anticipate chronological age.

Table 7 shows that the highest accuracy for the first group was 98.349%, while the minimum accuracy for the third group was 97.745%. The highest precision value was achieved for the third group at 98.924%, whereas the minimum value for the second group was 96.536%. For the F-score quantity, the highest value is for the first group at 98.782%, and the minimum is for the third group at 97.835%.

Table 8 shows that the proposed model accurately predicted 2455 out of 2480 samples for the first group, including ages 4–19, and mispredicted 25 images. For the second group, 20–29, the developed approach estimated 2874 images out of 2942 precisely while mispredicting 68 samples. A total of 1705 samples out of 1802 were estimated correctly, and 97 images were misestimated in the third group. For the fourth group, there are 3243 images, and 3198 samples were adequately predicted. In the last group, the presented algorithm correctly estimated 1593 images out of 1683. These outstanding results can open a new direction for deploying Artificial Intelligence (AI) in various domains.

From the conducted analysis and the obtained outcomes, the colors of the eyes make no difference to the age prediction, as the proposed model deals with their intensities and utilizes their values of peaks and bottoms. These values determine the age estimation but not the actual colors. Moreover, the presented algorithm can be deployed in various medical domains to diagnose diseases that are believed to be related to age, such as Alzheimer’s disease, Parkinson’s disease, and stroke, to treat or prepare suitable treatments.

## 5. Conclusions and Future Work

In this research, the proposed algorithm based on ensemble CNN to predict age was found to work. The model was trained, validated, and tested on the dataset downloaded from the Kaggle website. This dataset contains a large variety of ages from 4 to the late 50s. The results in the previous six cases indicate that the developed approach can produce promising outputs. In addition, it outperforms the implemented works referenced in the literature, and the average accuracy is approximately 97.3%.

Future work related to this study will minimize both performance metrics, MSE and MAE, to achieve greater accuracy. In addition, utilizing the human gait with a focus on the movement of arms will be explored for its potential as a feature that can be used to predict age.

## Figures and Tables

**Table 1:** Information on the training dataset.

Group#	Considered Ages	No. of Images	Dimension Size (H × W)
Group-1	4–20 years	24,790	227 × 227
Group-2	21–29 years	67,334	227 × 227
Group-3	30–39 years	38,157	227 × 227
Group-4	40–49 years	27,970	227 × 227
Group-5	50–59 years	30,749	227 × 227

**Table 2:** The scenario of the 5-fold cross-validation method.

	Fold-1	Fold-2	Fold-3	Fold-4	Fold-5
Run-1	Test	Train	Train	Train	Train
Run-2	Train	Test	Train	Train	Train
Run-3	Train	Train	Test	Train	Train
Run-4	Train	Train	Train	Test	Train
Run-5	Train	Train	Train	Train	Test

**Table 3:** Actual ages of applied inputs.

Case Number	Actual Age	Gender
Case 1	26	Female
Case 2	26	Female
Case 3	32	Male
Case 4	46	Male
Case 5	50	Male
Case 6	5	Female

**Table 4:** Calculated MSE and MAE.

Evaluated Performance Metrics	Obtained Values
MSE	1.166
MAE	0.833

**Table 5:** Performance metrics values.

Evaluated Performance Metrics	Obtained Values
TP	11,825
FP	167
FN	158
Accuracy	97.32%
MSE	±8.69 years
MAE	±2.43 years
Precision	98.607%
Recall	98.681%
F-score	98.644%

**Table 6:** The assessment results.

Work Conducted	Accuracy	MSE	MAE
Obday, H., et al. 2022 [3]	Not mentioned	Not mentioned	13.05 years
Ramirez, J.G., et al. 2022 [4]	Not mentioned	Not mentioned	2.57 years
Cao, Y., et al. 2022 [5]	Not mentioned	Not mentioned	4.13 years
Odion, P.O., et al. 2022 [6]	95%	Not mentioned	Not mentioned
Gowroju, S., et al. 2022 [7]	96%	Not mentioned	Not mentioned
Hassan, M., et al. 2021 [8]	86.9%	Not mentioned	6.44 years
Baecker, L., et al. 2021 [9]	Not mentioned	Not mentioned	3.7–4.7 years
Shigueoka, L.S., et al. 2021 [12]	96.2%	Not mentioned	Not mentioned
Dalrymple, K.A., et al. 2019 [18]	90%	Not mentioned	Not mentioned
The Proposed algorithm	97.32%	8.69 years	2.43 years

**Table 7:** The achieved performance results among different ages.

Categories of Ages	Accuracy	Precision	F-Score
4–19	98.349%	96.792%	98.782%
20–29	97.891%	96.536%	98.664%
30–39	97.745%	98.924%	97.835%
40–49	98.021%	97.896%	97.991%
50–59	97.892%	98.794%	98.476%

**Table 8:** The resultant confusion matrix of the presented algorithm.

**Prediction groups**	**Actual Groups**					
		4–19	20–29	30–39	40–49	50–59
	4–19	2455	7	4	9	5
	20–29	23	2874	19	11	15
	30–39	12	35	1705	27	23
	40–49	7	18	13	3198	7
	50–59	28	17	20	25	1593

## Data Availability

The dataset utilized in this study was downloaded from the Kaggle website and is available upon request.
